# Single-layer silicon metalens for broadband achromatic focusing and wide field of view

**DOI:** 10.1038/s41598-025-27208-1

**Published:** 2025-12-08

**Authors:** Jian Cao, Sarra Salhi, Jonathan Peltier, Jean-René Coudevylle, Samson Edmond, Cédric Villebasse, Etienne Herth, Laurent Vivien, Carlos Alonso-Ramos, Daniele Melati

**Affiliations:** Centre de Nanosciences et de Nanotechnologies, Université Paris-Saclay, CNRS, 91120 Palaiseau, France

**Keywords:** Engineering, Optics and photonics, Physics

## Abstract

Achieving simultaneous broadband achromatic focusing and a wide field of view remains a significant challenge for metalenses. In this work, we begin with a quadratic phase profile, enabling full field-of-view designs, and apply dispersion engineering to minimize variations of the focal length across wavelengths, thereby substantially reducing both longitudinal and transverse chromatic aberrations. This is accomplished using only the propagation phase in a single layer of waveguide-like rectangular meta-atoms, without relying on geometric phase contributions. The fabricated metalens experimentally demonstrates a field of view of $86^{\circ }$, along with a tenfold reduction in focal length variations with wavelength compared to a conventional quadratic metalens, achieving a measured relative shift as low as 1.3% across the 1.5 µm - 1.6 µm range (limited by our experimental setup). This improvement also results in a constant focusing efficiency in the considered wavelength range, where a reference quadratic metalens exhibits a nearly twofold reduction. These experimental results validate the effectiveness of our design strategy in simultaneously enhancing the operational bandwidth and field of view of metalenses. The demonstrated performance can directly benefit beam steering applications in the near-infrared wavelength range and provides a path toward achromatic, wide field-of-view metalenses in the visible range for imaging systems.

## Introduction

Metasurfaces are nano-structured surfaces realized by arranging meta-atoms at sub-wavelength distances^1–3^. Depending on the design of the meta-atoms and their arrangement, metasurfaces can be used to implement a variety of traditional optical functionalities, such as lensing^4^, polarization manipulation^5^, beam steering^6,7^, or holographic image projection^8^. The possibility to replace bulk optical lenses with metasurfaces (also referred to as metalenses) is of interest for a range of applications, e.g. imaging, LiDAR, or free space communication devices, where the reduction of the system footprint is paramount in achieving scaling and widespread usage. Demonstrating metalenses with sufficiently high performance is however crucial. Specifically, operation over a broadband wavelength range and a wide field of view have been standing challenges for metalenses, especially when the two need to be obtained simultaneously.

Chromatic aberration in metalenses depends on the optical dispersion of the meta-atoms and several approaches have been proposed to correct it and achieve an achromatic behavior^3,9–25^. Multi-layer metalenses, possibly using meta-atoms with different heights and combined with phase plates, can be used to largely reduce chromatic aberration while maintaining a high focusing efficiency^16,25^, at the cost of high fabrication complexity. As an example, by exploiting this method, Balli et al.^16^ demonstrated a hybrid achromatic metalens working from 1.0 µm to 1.8 µm wavelength with an average focusing efficiencies of 60% (computed with respect to the incident power on the metalens). Alternatively, chromatic aberration can be compensated by carefully engineering the phase and dispersion across a single-layer metalens in order to ensure that the focal distance of the metalens remains constant despite the frequency change. This approach has been demonstrated by tailoring the geometric parameters of resonant or waveguide meta-atoms and also combining meta-atom shape tuning with geometric phase control^26^. Chen et al.^4^ demonstrated an achromatic metalens working in the visible range with a relative focal shift of 9.3% over a 200 nm wavelength range around a central wavelength of 530 nm. By exploiting a similar approach while also engineering the dispersion of the target phase profile, Shrestha et al.^13^ demonstrated an achromatic metalens with a relative focal shift of 2% over a 200 nm wavelength range around a working wavelength of 1.6 µm.

Besides chromatic aberration, metalenses focusing performance can also be severely affected by off-axis aberration. For example, a metalens with a hyperbolic phase profile has a high focusing quality for on-axis wavefronts. However, focusing capabilities rapidly degrade even for moderately tilted illumination^27,28^. The problem of designing wide field-of-view metalenses has hence been extensively explored in the literature^28–39^. Placing an aperture stop in front of a metalens can help increase its field of view^40^, but it also largely reduces transmission efficiency. Arrays of metalenses^34^, where each metalens is optimized to focus light from different angles, have also been exploited for enlarged fields of view. This method, however, increases footprint, design, and fabrication complexity. Likewise, doublet metalenses or multilayer metalenses^30,41^ can achieve a large field of view, requiring however complex fabrication processes. Finally, a singlet metalens can achieve full field of view thanks to judiciously designed phase profiles, e.g., modified hyperbolic phase profiles based on optimized polynomials, spherical phase profiles^27^, or more commonly quadratic phase profiles^20,28,31,32^. This approach usually trades off a wider field of view with a reduced focusing efficiency compared to the theoretically achievable limit^42^ but ensures at the same time a relatively simple fabrication process and a low design complexity.

**Fig. 1:**
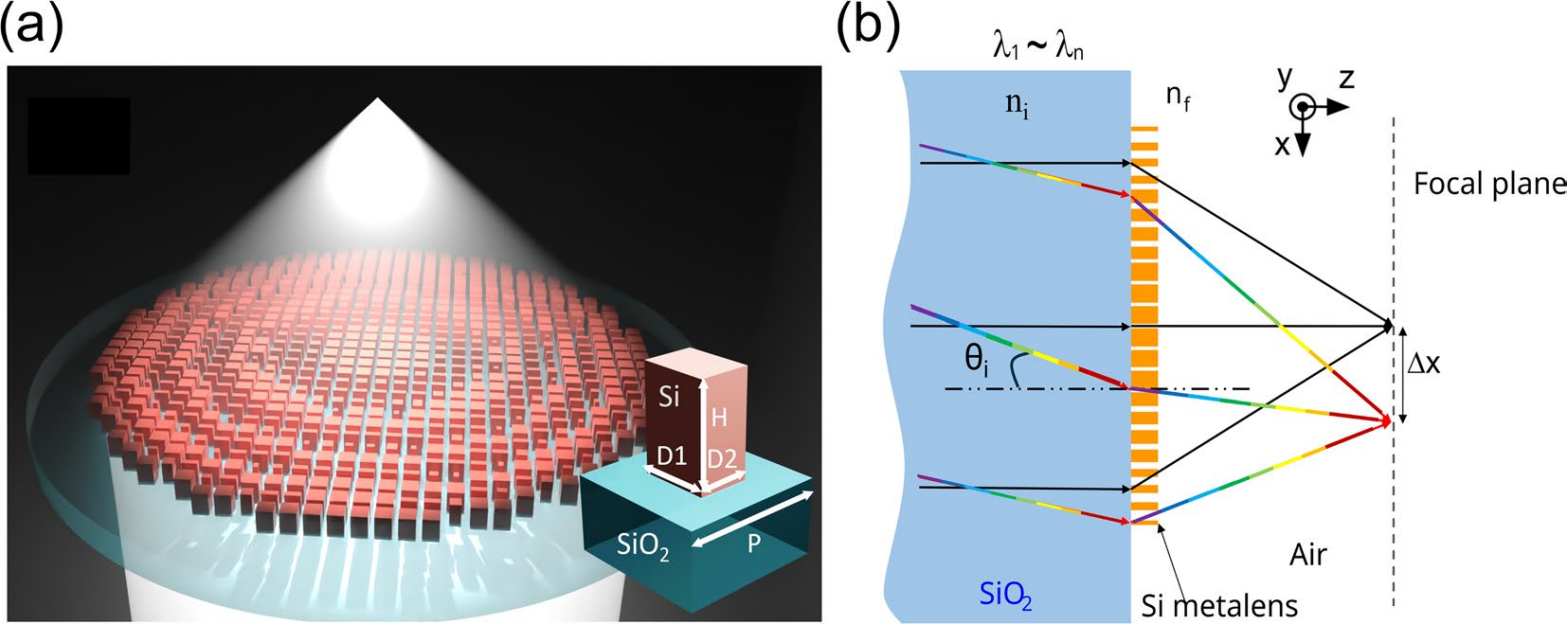
Achromatic and wide field of view metalens. (**a**) Pictorial representation of the metalens operation. In the inset, a sketch of the silicon meta-atoms is used to realize the metalens. The height is H = 700 nm. The sizes D1 and D2 vary from 150 nm to 500 nm with a period p = 650 nm. (**b**) Representation of the desired functionality. Longitudinal chromatic aberration is minimized and the focal plane distance from the metalens is maintained constant within a given wavelength range. At the same time, when the incident light is tilted of an angle $\theta _{i}$ in the x-z plane, the focal distance does not change, and the focal spot shifts of an amount $\Delta x$ along the x-axis (wide filed of view). Variations of $\Delta x$ with wavelength (transversal chromatic aberration) are minimized.

One possible solution to design a metalens that achieves at the same time achromatic focusing and a wide field of view consists of engineering the dispersion of a base phase profile chosen for its wide field of view. Previous designs reported by Xu et al.^43^ required the use of geometric phase to implement a quadratic phase profile using meta-atoms with different rotations and then controlled the phase profile dispersion exploiting the propagation phase within the waveguide-like meta-atoms. Simulation results showed a field of view up to $160^{\circ }$ working in the 3.8 - 4.2 µm wavelength range. Relying on a geometric phase contribution, the metalens required left-handed circularly polarized incident light. With a similar approach, Yu et al.^44^ combined two metalenses, one based on the propagation phase and the second on the geometric phase, to design a doublet metalens with a field of view of $68^{\circ }$ working on a 640 nm - 820 nm wavelength range (simulation results). Moon et al.^45^ recently proposed the design of a dual-layer metalens implementing a broadband quadratic phase profile exploiting only a propagation phase delay achieving (in simulations) a field of view of $80^{\circ }$ in the 450 nm - 635 nm, with relative focal distance shifts lower than 2%. Alternatively, without starting from a pre-defined phase profile, Yang et al.^46^ combined a direct search algorithm with a deep learning model to design free-form meta-atoms and exploit their diverse dispersion behaviors to directly optimize the design of metalens with a field of view of $160^{\circ }$ working on a 1.0 µm - 1.2 µm wavelength range (simulation results).

In this work, we experimentally demonstrated a singlet achromatic and wide field-of-view metalens designed by matching the dispersion of a quadratic phase profile used as a base. We demonstrated that we can achieve this goal without relying on multi-layer structures or a geometric phase contribution but solely using the propagation phase of a single layer of waveguide-like silicon meta-atoms, hence removing the requirement for circularly polarized light. Experimental characterizations of the metalenses showed a field of view of $86^{\circ }$ combined with a ten-fold reduction in the dependence of the focal distance on wavelength compared to a reference quadratic metalens without dispersion engineering. The measured relative focal shift was as low as 1.3% in the 1.5 µm - 1.6 µm wavelength range, limited by our available setup. The metalens also exhibited a constant focusing efficiency with wavelength, a sharp improvement compared to the reference quadratic metalens which showed nearly a twofold reduction in focusing efficiency over the same wavelength range. As shown in Table S1 of the supplementary information document, this is, up to our knowledge, the best experimental performance for an achromatic and wide field-of-view singlet metalens reported so far in the literature.

## Design

**Fig. 2:**
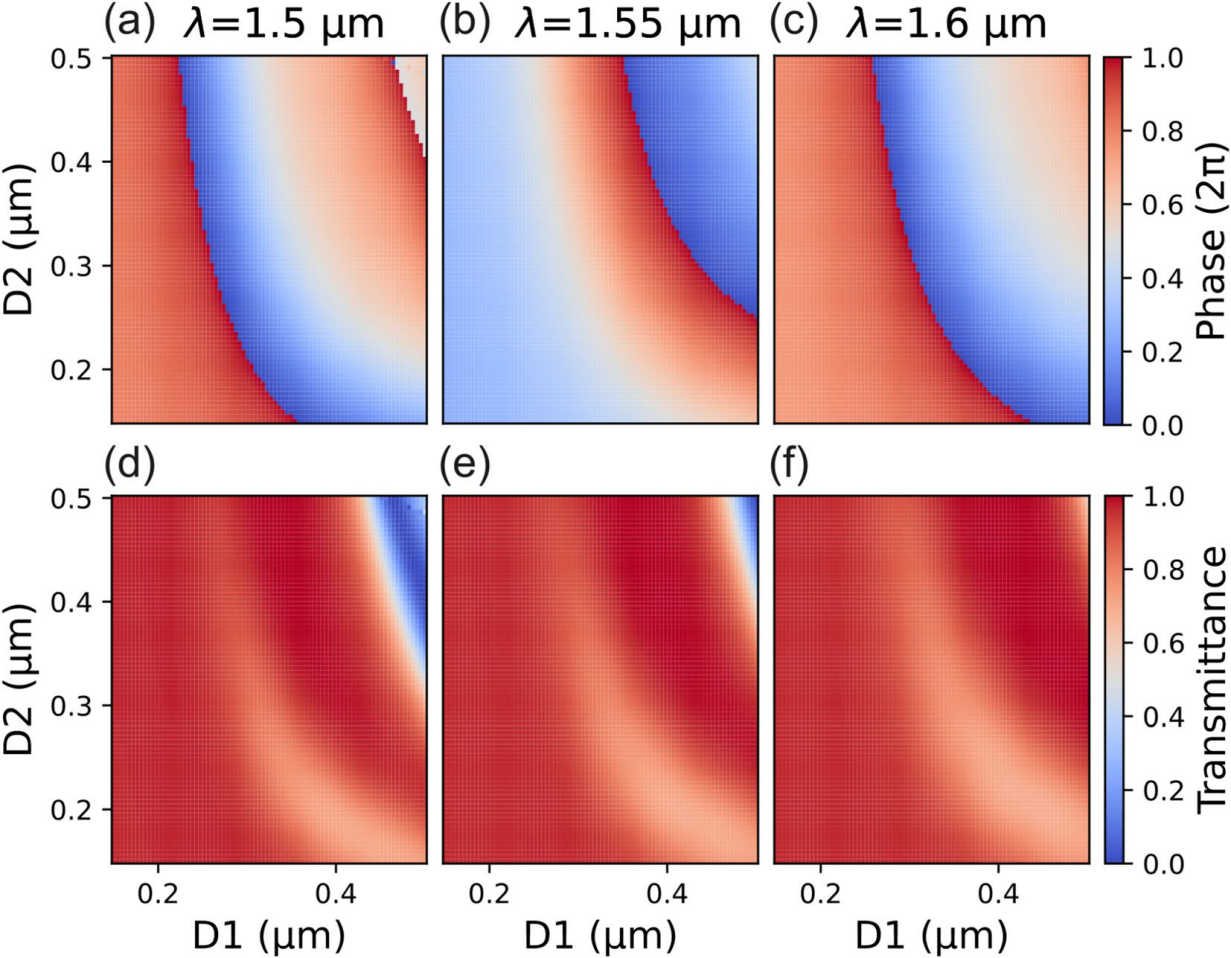
Phase delay and transmission efficiency of the meta-atoms. (**a**)-(**c**) Map of the phase delay imparted by meta-atoms with D1 and D2 varying from 150 nm to 500 nm and with a period of 650 nm, for three different wavelengths in the 1.5 µm - 1.6 µm range. In the simulations, light propagates from the SiO$_2$ substrate toward the meta-atom. For each wavelength, the meta-atoms phase delay covers a 2$\pi$ range. (**d**)-(**f**) Corresponding transmission efficiency maps for the same series of meta-atoms. Most meta-atoms have an efficiency higher than 0.5 for the three wavelengths.

For the design of the metalens we considered 700-nm-thick silicon pillars with a rectangular shape placed on a SiO$_2$ substrate, as shown in Fig. 1(a). We exploited rigorous coupled-wave analysis (RCWA)^47^ to simulate the behavior of the meta-atoms, assuming a locally periodic structure^17,48^. This approximation holds well for waveguide-like meta-atoms^11^. We also assumed the incident light was TE-polarized. We built a library of available meta-atoms by sweeping the sizes D1 and D2 of the pillar from 150 nm to 500 nm while keeping the meta-atoms period fixed at 650 nm. In the sweeps, we limited the smallest feature (silicon pillars and pillar gaps) to be larger than 150 nm to improve fabrication reliability. We computed the phase delay and transmission efficiency imparted by each meta-atom shape to a plane wave propagating orthogonally to the silicon pillars for five different wavelengths in the 1.5 µm - 1.6 µm range. The choice of this wavelength range was limited by our experimental capabilities and not by the design approach itself. The use of five wavelength points ensured a robust sampling of the meta-atoms dispersion, as can be seen in Fig. S1 of the supplementary information document reporting on the phase delay dependence on wavelength for a handful of meta-atoms chosen as examples. Simulation results are shown in Fig. 2 as a function of D1 and D2 for $\lambda$ = 1.5 µm, $\lambda$ = 1.55 µm, and $\lambda$ = 1.6 µm. Most of the meta-atoms efficiencies are higher than 50% while their phase delay can cover the required 2$\pi$ range across the entire wavelength range.

The design of the metalens was based on two objectives, as shown in Fig. 1(b). First, light coming at different angles needed to be focused on the same focal plane, with a fixed focal distance (wide field of view). At the same time, light at different wavelengths within the 1.5 µm - 1.6 µm wavelength range needed to be focused at the same focal spot, whose position was dictated by the angle of incidence (low longitudinal and transverse chromatic aberrations). We chose as a starting point for the design a quadratic phase profile which had already been demonstrated enabling focusing over a 180-degree field of view^28,31,32,37^:

1 $$\begin{aligned} \begin{aligned} \phi (r, \lambda )\ &=\ -\ k\frac{r^2}{2f}\ =\ -\frac{\pi n_{f} r^2}{\lambda f}. \end{aligned} \end{aligned}$$

In (1), $\phi$(r, $\lambda$) is the phase delay imparted by the metalens, which depends on the radial position r and wavelength $\lambda$, $k=n_fk_0$ is the wave-number in the propagation medium, $k_0$ is the wave-number in vacuum ($k_0=2\pi /\lambda$), $n_{f}$ is the refractive index in the focusing region, and *f* is the designed focal length.

As schematically represented in Fig. 1(b), with a quadratic phase profile the focal spot shifts along the focal plane upon tilting of the incident beam. Assuming the incident beam is propagating along the z-axis and it is titled in the x-z plane with an angle $\theta _i$ from the normal to the metalens surface, the phase of the wavefront after the metalens is

2 $$\begin{aligned} \phi (r, \lambda )\ &=\ \phi _0\ -\ k\frac{r^2}{2f}\ -\ kxn_isin\theta _i \nonumber \\&=\ \phi _0\ -\ \frac{\pi n_{f}}{\lambda f}(x^2 + y^2)\ -\ \frac{2\pi n_in_{f}}{\lambda }xsin\theta _i \nonumber \\ \ &=\ \phi _0\ -\ \frac{\pi n_{f}}{\lambda f}[(x + n_i f sin\theta _i)^2 + y^2]\ +\ \frac{\pi n_{f} }{\lambda }n_i^2 f sin^2\theta _i \end{aligned}$$

where $n_i$ is the refractive index of the incident region. As shown in Eq.(2), a tilt $\theta _i$ in the x-z plane introduces a spatial shift of the focal spot of $\Delta x = -n_ifsin\theta _i$ along the x-axis. The same is true for tilts in the orthogonal y-z plane, which causes a shift of the focal point along the y-axis. It should be noticed, however, that the phase delay in Eq. (1) imparted by the metalens depends on the wavelength $\lambda$ of the incident wavefront, causing the focal spot to move in the longitudinal direction z, i.e., changing the focal distance (longitudinal chromatic aberration). This also causes at the same time a shift of the focal spot in the transversal direction for tilted illumination (transversal chromatic aberration) since $\Delta x$ depends on wavelength via the focal distance *f*, as can be seen in Eq. (2).

**Fig. 3:**
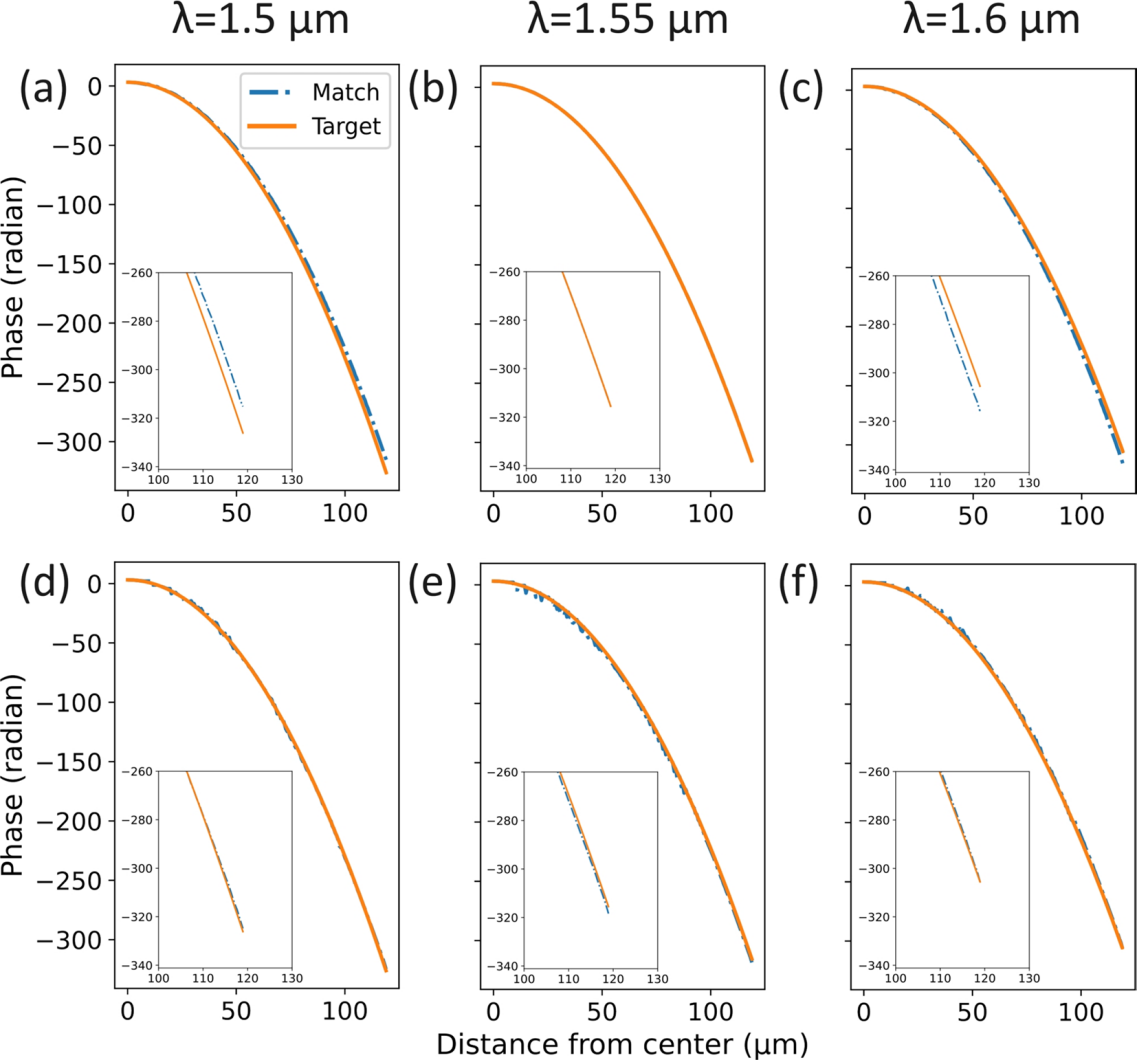
Metalens phase profile matching. (**a**)-(**c**) Phase profile for a reference single-wavelength design metalens at three different wavelengths in the 1.5 µm - 1.6 µm range. At each position in the metasurface, we chose the meta-atom whose phase delay matched with the target value at $\lambda$ = 1.55 µm. The solid orange line is the target phase profile while the blue dotted line is the resulting matched phase profile. (**d**)-(**f**) Phase profile for the broadband design metalens for the same series of wavelengths. Instead of matching the required phase at only one wavelength, we chose the meta-atom which minimized the average phase error over five different wavelengths in the 1.5 µm - 1.6 µm range.

In order to compensate for chromatic aberrations, we chose for each position in the metalens the element in the meta-atom library that minimized the difference between the target phase delay at that location and the actual phase delay of the meta-atom, averaged over five wavelengths in the 1.5 µm - 1.6 µm range:

3 $$\begin{aligned} \begin{aligned}&(D_1(r), D_2(r))\ =\ (D_1,D_2)|_{min{\{\Delta \Phi \}}}\\&with\ \Delta \Phi \ =\ E_i\{\Phi _{meta}(\lambda _i, r)\ -\ \Phi _{target}(\lambda _i,r)\} \end{aligned} \end{aligned}$$

where $D_1$ and $D_2$ are the meta-atom sizes, *r* is again the radial position from the center of metalens, $\lambda _i$ is the chosen wavelength, $\Delta \Phi$ is the difference between the meta-atom phase delay $\Phi _{meta}$ and the target phase $\Phi _{target}$, and $E_i$ denotes the average over wavelength. This approach allows placing at each location in the metalens the meta-atom whose phase delay and dispersion match in the best possible way those of the target phase profile within the considered wavelength range, ensuring a fixed focal distance. Although design is optimized at five wavelength points, this is sufficient to achieve a reliable match of the phase profile over the continuous 1.5 µm - 1.6 µm wavelength range. Throughout the manuscript, we refer to the metalens designed with this approach as the *broadband design*. As a reference, we also designed a regular wide field of view metalens operating at one single wavelength by matching the metalens phase profile only with the target quadratic phase at $\lambda$ = 1.55 µm. We refer to this as the *single-wavelength design*.

Figure 3 shows the obtained phase profile for a metalens with a diameter of 240 µm and a focal distance *f* = 90 µm for both broadband and single-wavelength designs at three different wavelengths (dot-dashed blue lines). The figure also reports the quadratic phase profile that is required at each wavelength to obtain the desired focal distance of 90 µm (target profile, solid orange lines). For the single-wavelength design, the obtained phase profile matches well with the target requirement at $\lambda$ = 1.55 µm, but considerable deviations can be seen for the other wavelengths, see Fig. 3(a-c). On the contrary, for the broadband design the obtained phase matches well with the target across the entire 1.5 µm - 1.6 µm spectrum, even if some additional noise can be seen around the center, Fig. 3(d-f). This noise has an impact on the focusing performance, as discussed in Section Analysis. Figure S2 of the supplementary information document reports directly the difference between the target and matched phase as a function of wavelength and position in the metalens for the two designs. Figure S3 reports instead the comparison between target and matched phase profiles at multiple wavelength points beside those used for the design, demonstrating a good match over the continuous 1.5 µm - 1.6 µm wavelength range.

## Experimental results

**Fig. 4:**
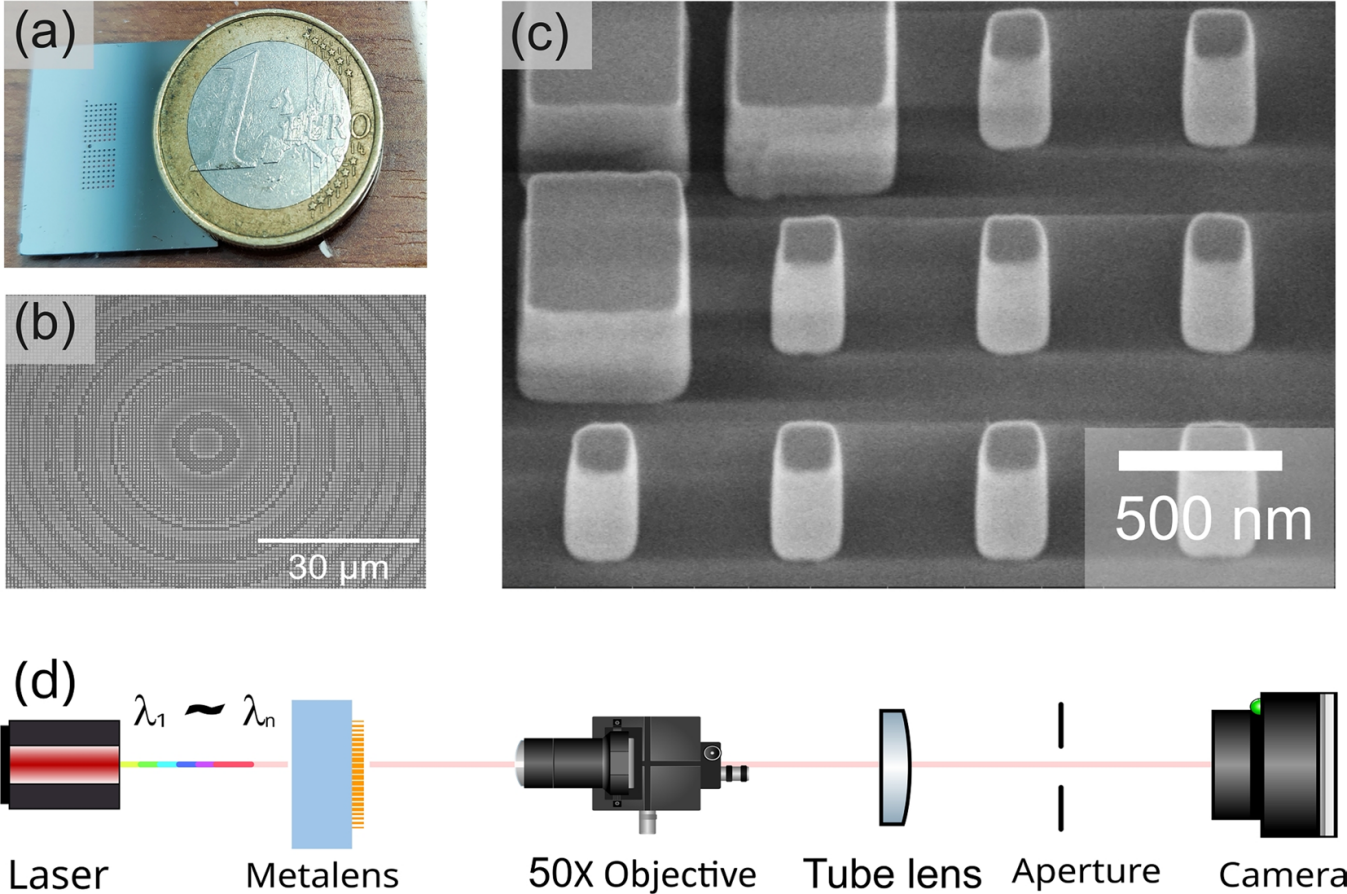
Fabricated metalenses. (**a**) Optical image of a fabricated chip with several test metalenses. (**b**) An SEM image of the central part of one of the metalenses and (**c**) a zoom-in on the silicon meta-atoms. The smallest pillars are 150 nm x 150 nm in size. (**d**) Schematic of the experimental setup used for the characterization of the metalenses. The wavelength of the laser source could be tuned in the 1.5 µm - 1.6 µm range. The sample and imaging system could be rotated with respect to the source up to $90^{\circ }$. The focal spot was imaged using a camera with a 50X objective and corresponding tube lens.

Metalenses were fabricated using electron beam lithography and RIE - ICP etching (see Method). Optical and scanning electron microscope images of the fabricated devices are reported in Fig. 4(a-c). For both the broadband and the reference single wavelength designs, we fabricated different metalenses with numerical apertures NA of 0.74, 0.8, and 0.86, and a constant focal length of 90 µm. The setup shown in Fig. 4(d) was used to characterize the focal spot, the field of view, and the focal length of the fabricated metalenses (see Method). The laser beam passed through the metalens and the focal spot was imaged on the camera through a 50X objective and corresponding tube lens. The wavelength of the laser was tuned between 1.5 µm and 1.6 µm and the illumination angle could be varied from $0^{\circ }$ to $90^{\circ }$. The metalens position could be finely tuned through a piezoelectric stage. For the characterization of the focusing efficiency, a power meter was used instead of the camera.

**Fig. 5:**
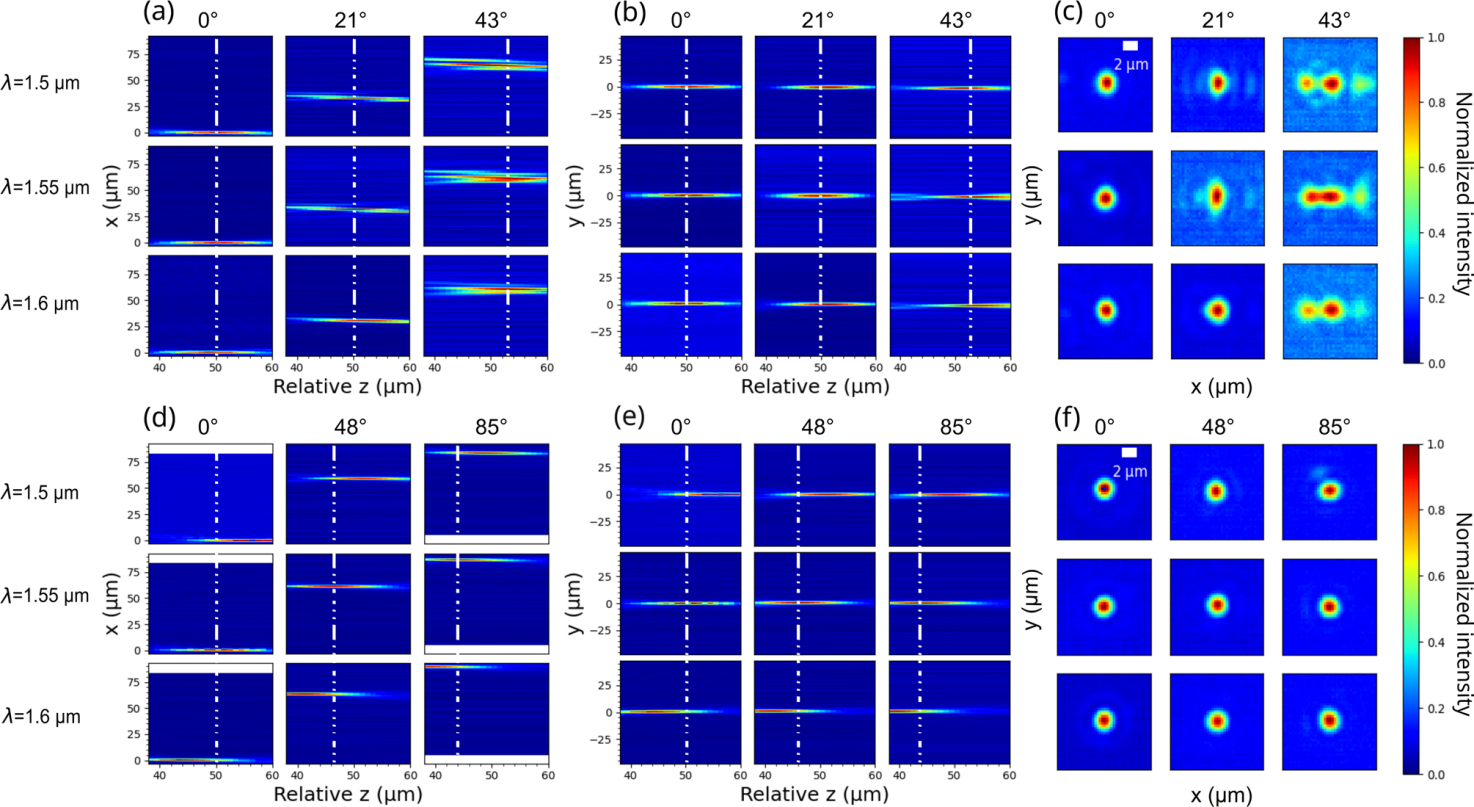
Experimental characterization of the metalens focusing. (**a**-**c**) Results for the broadband metalens with NA = 0.8 and *f* = 90 µm at $\lambda$ = 1.5 µm, $\lambda$ = 1.55 µm and $\lambda$ = 1.6 µm. Panel (**a**) shows a cross-section of the focal spot in the x-z plane while (**b**) in the y-z plane, as defined in Fig. 1(b), with tilted incidence in in x-z plane from $0^{\circ }$ to $43^{\circ }$. The white dashed lines mark the center of the focal spot at $\lambda$ = 1.55 µm. (**c**) Image of the focal spot in the x-y plane. (**d**)-(**f**) The same series of results for the single-wavelength metalens with NA = 0.8, and f = 90 µm. The incident angle varies from $0^{\circ }$ to $85^{\circ }$. The blank areas appearing in (**d**) were areas outside of the detector aperture which are maintained to ensure the axes are consistent across the figures.

Figure 5 shows the characterization of the focal spots of metalenses with NA = 0.8 and focal distance f= 90 µm at three different wavelengths in the 1.5 - 1.6 µm range and three different incident angles. The white dashed lines mark the focal spot position at $\lambda$ = 1.55 µm along the propagation axis z. Figure 5 (a-c) refer to the broadband metalens, with incident angles of $0^{\circ }$, $21^{\circ }$, and $43^{\circ }$. Figure 5 (d-f) report on the results for the single wavelength reference metalens with incident angles of $0^{\circ }$, $48^{\circ }$, and $85^{\circ }$. Figure 5 (a,d) show a cross-section of the focusing pattern in the x-z plane, Fig. 5 (b,e) in the y-z plane, and Fig. 5 (c,f) in the x-y plane at the center of the focal spot. Figure S5 in the supplementary information document reports x-y cross-sections taken at a fixed distance from the metalens equal to the focal distance at $\lambda$ = 1550 nm. As expected and discussed in Design section, both broadband and single wavelength metalenses show a transversal shift along the x-axis when the incident beam is tilted in the x-z plane. The broadband metalens has a field of view limited to $\pm 43^{\circ }$ after which the focusing spot becomes severely degraded. This is due to the increased phase noise that is introduced during the phase profile matching for the broadband design compared to the single wavelength one, as discussed in details in Analysis section. The full width at half maximum of the focal spot measured along the x-axis remains almost constant (1.61 µm - 1.94 µm, Fig. S4 of the supplementary information document) until the illumination angle approaches $43^{\circ }$. Focusing performance degenerates sharply at $43^{\circ }$ (FWHM = 8.07 µm) and beyond this angle a focal spot could not be observed anymore. On the contrary, the single-wavelength metalens exhibit as expected a nearly full field of view (close to $\pm 90^{\circ }$), with almost no off-axis aberrations. However, in the considered wavelength range, the single-wavelength metalenses show a strong chromatic aberration, and the focal distance changes noticeably as the wavelength varies for all incident angles. For the broadband metalens, instead, the shift of the focal spot is largely reduced over the same wavelength range and for all the considered tilting angles.

**Fig. 6:**
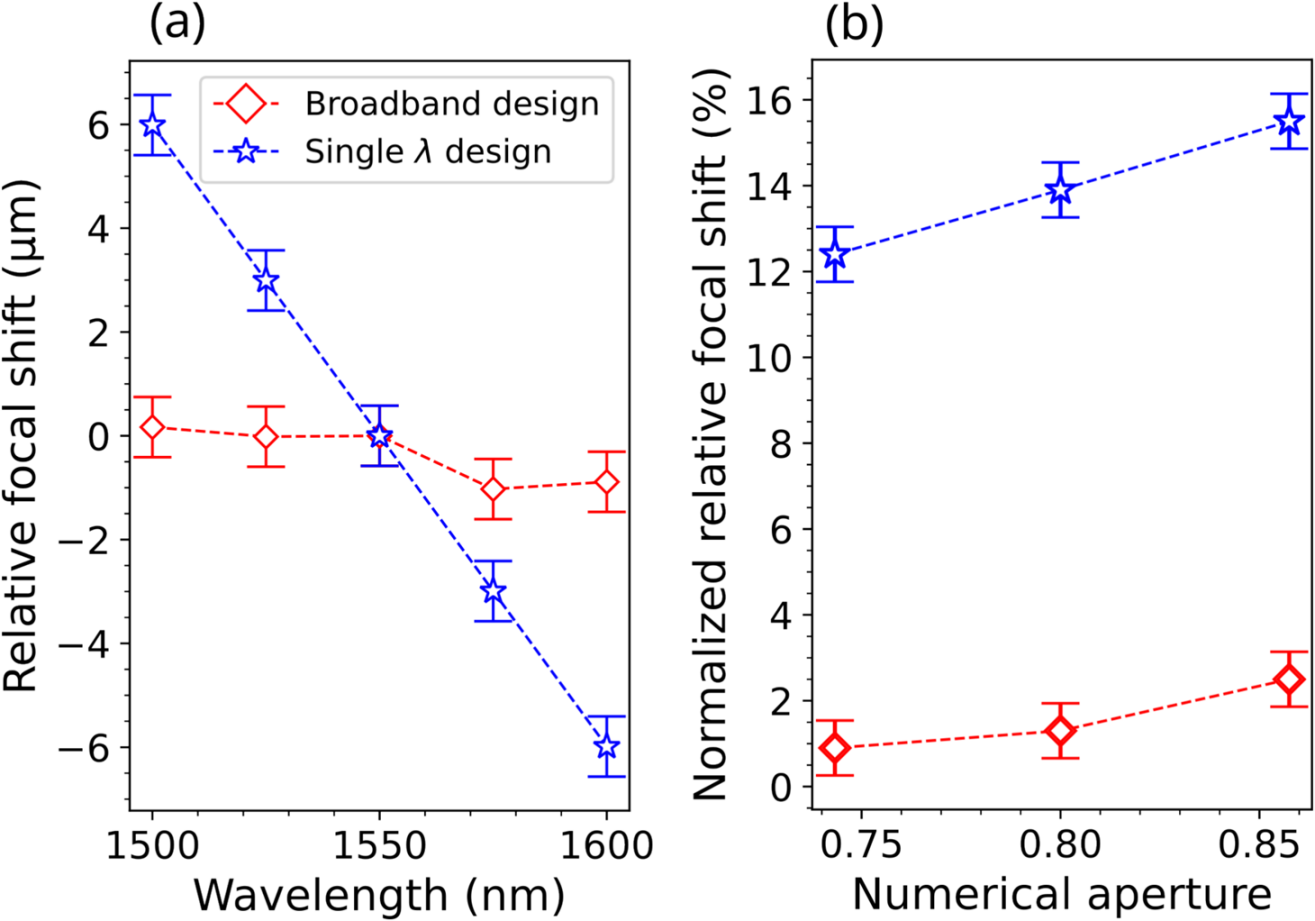
Performance of the broadband and single wavelength metalens under normal illumination. (**a**) Relative shift of the focal distance at $\lambda$ = 1.55 µm for the broadband design (red diamond markers) and the single-wavelength metalens (blue star markers). (**b**) The normalized relative focal shift across the 100 nm bandwidth for the broadband design (red line) and the single-wavelength metalens (blue line).

A more detailed analysis of the behavior of the two metalenses for normal illumination ($0^{\circ }$ tilting) is shown in Fig. 6. In particular, Fig. 6(a) reports on the variation of the focal distance as a function of the wavelength (relative focal shift) for broadband and single wavelength metalenses with NA = 0.8 and *f* = 90 µm. The error bar is calculated as $\Delta u=\Delta \sigma / \sqrt{3}$, where $\Delta u$ is the measurement error, $\Delta \sigma$ is the experimental uncertainty of the setup on distances measured along the propagation axis, conservatively estimated as 1 µm, and $\sqrt{3}$ comes from the assumption that error distribution is uniform. It can be seen that for the broadband design, the longitudinal chromatic aberration was largely compensated across the considered 100 nm wavelength bandwidth, with the relative focal shift changing less than 1.2 µm, i.e., 1.3 % compared to the designed focal length. On the contrary, for the single-wavelength metalens, the relative focal shift exhibits a linear dependence on wavelength with a variation of 12.0 µm across the 100 nm bandwidth, ten times larger than the broadband metalenses. Figure 6(b) shows the dependence on the numerical aperture of the normalized relative focal shift, i.e., the ratio between the relative focal shift across the 100 nm bandwidth and the designed focal length. For the broadband design, the normalized relative focal shift remains smaller than 2 % for numerical apertures between 0.75 and 0.85. For the regular single wavelength design, the normalized relative focal shifts are comprised between 12 % at NA = 0.75 and 15 % at NA = 0.85. For both broadband and single-wavelength designs, the normalized relative focal shift tends to worsen as the numerical aperture increases. This could be explained by the fact that the achromatic bandwidth decreases for large numerical apertures^12,49^.

**Fig. 7:**
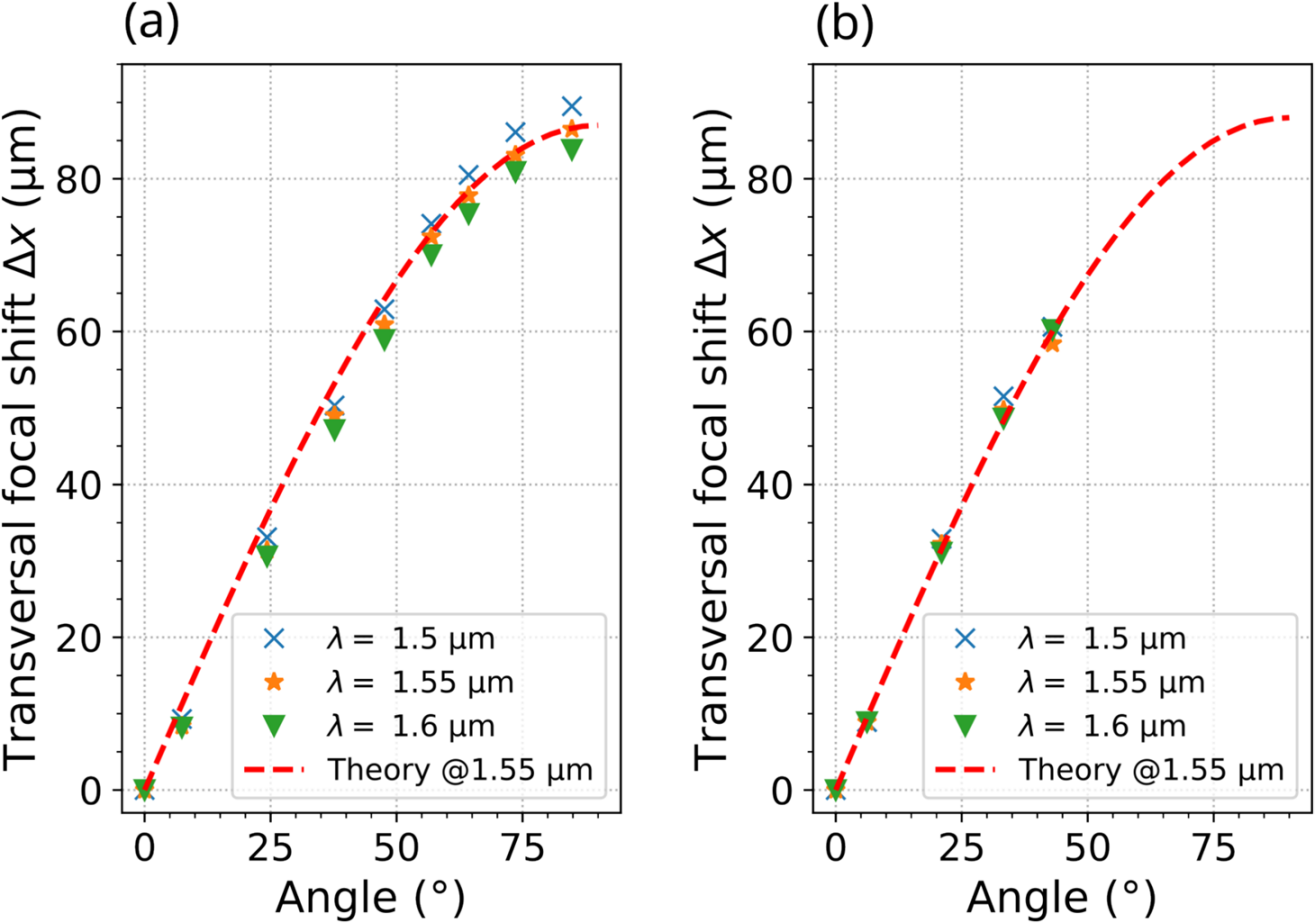
Focal shift under tilted illumination. Figures report on the transversal focal shifts along the x-axis when the incidence angle is titled in the x-z plane for three different wavelengths in the 1.5 µm - 1.6 µm range, for (**a**) the single wavelength metalens and (**b**) the broadband design. Red dashed lines show the theoretical shift obtained by fitting results at $\lambda$ = 1.55 µm.

The characterization of the single wavelength and broadband metalenses under tilted illumination is reported in Fig. 7 which shows the shift of the focal spot along the transversal direction x as a function of the incident angle for $\lambda$ = 1.5 µm, $\lambda$ = 1.55 µm and $\lambda$ = 1.6 µm. The transversal focal shift is calculated with respect to the position of the focal spot for an incident angle of $0^{\circ }$. As expected, as the incident angle increases, the single wavelength design shows larger variations of the transversal focal shifts across the 100 nm bandwidth compared to the broadband design (transversal chromatic aberration). For the single wavelength metalens, the difference in the transversal focal shift between $\lambda$ = 1.5 µm and $\lambda$ = 1.6 µm is 2.5 µm when the incident angle is $24^{\circ }$ and it increases to 4.0 µm when the incident angle is $48^{\circ }$. For the broadband design, the difference is 1.8 µm when the incident angle is $21^{\circ }$ and it only slightly increases to 2.2 µm when the incident angle increases to $43^{\circ }$, demonstrating an effective compensation also of the transversal chromatic aberration in the broadband design. As a reference, red dotted lines report on the theoretical focal shift $\Delta x=-n_ifsin\theta _i$ (as derived from Eq. (2)) obtained by fitting the experimental results at $\lambda$ = 1.55 µm. The fitted focal distances at this wavelength for the single wavelength and broadband designs were 87 µm and 88 µm, in good agreement with the designed focal length of 90 µm.

**Fig. 8:**
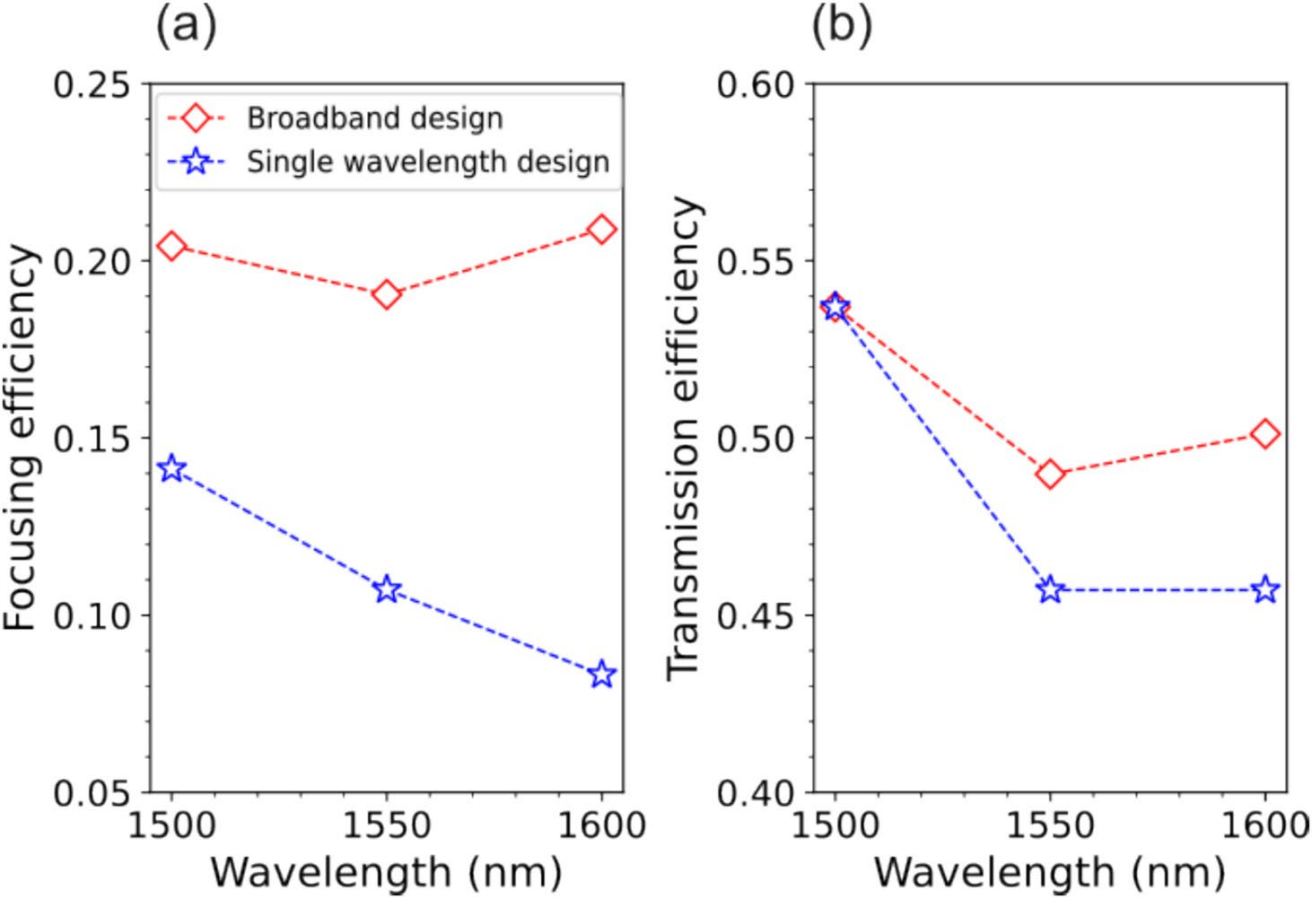
Metalenses efficiency. (**a**) Focusing efficiency at normal incidence for the broadband (red line with diamond markers) and single-wavelength metalenses (blue line with star markers) with NA = 0.8 in the 1.5 µm - 1.6 µm wavelength range. (**b**) Transmission efficiency for the same metalenses.

Lastly, we characterized the focusing and transmission efficiencies of both single wavelength and broadband metalenses, considering again NA = 0.8 and *f* = 90 µm, over the considered 100 nm bandwidth and under normal illumination. For both metalenses, the imagining plane was maintained fixed during the measurements at the focal distance for $\lambda$ = 1500 nm. Focusing efficiency was defined as the energy in the x-y plane integrated within a circle three times larger than the focal spot radius divided by the background signal where no metalens was present (but just the unpatterned substrate). The characterization results as a function of the wavelength are reported in Fig. 8 (a). Transmission efficiency was instead defined as the ratio between the energy integrated across the entire metalens area and the background signal, reported in Fig. 8 (b). The broadband design (red dashed lines) shows a focusing efficiency that is almost independent of wavelength. In the 1.5 - 1.6 µm wavelength range, the focusing efficiency varies between 19.1% to 20.9%. Within the same range, the focusing efficiency decreases from 14.1% to 8.3% for the single wavelength metalens (blue dashed lines), an almost twofold reduction. This is a consequence of the fact that the single-wavelength design is affected by a larger chromatic aberration which cause energy integration to decrease as the focal spot moves out of the fixed imaging plane while wavelength is swept. The difference between the two designs is less marked for the transmission efficiency, that varies from 49.0% to 53.7% for the broadband design and from 45.7% to 53.7% for the single wavelength design.

Table S1 in the supplementary information document compares these results with other experimental performances reported in the literature for achromatic and wide field-of-view metalenses. The metalens described in this work has a focusing efficiency in line with previous results for metasurfaces based on quadratic phase profiles^31,32^. At the same time, it demonstrates the best performance for a singlet metalens in terms of combined normalized relative focal shift and field of view when compared to previously reported broadband achromatic^4,11,13,16,19^ and wide field of view metalenses^20,32,36,40^.

## Analysis

**Fig. 9:**
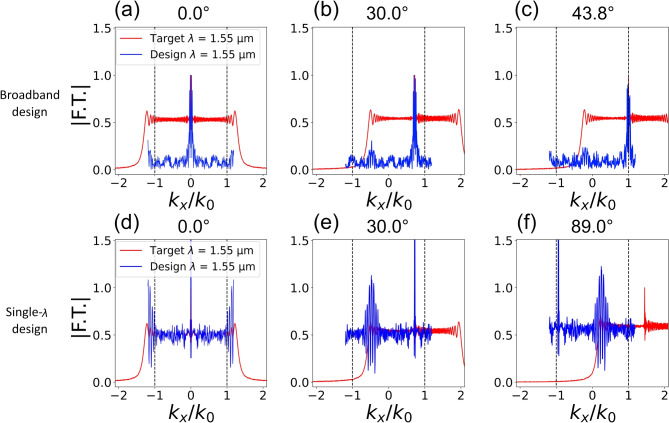
Fourier transform spectra of the electric field after the metalenses along the normalized $k_x$ axis. Results for (**a**)-(**c**) the broadband design and (**d**)-(**f**) the single-wavelength design are shown with blue solid lines for three different illumination angles. Results for an ideal metalens exactly implementing the target phase profile at $\lambda$ = 1.55 µm are shown with red solid lines. The black dotted lines mark the propagation region bounds in the normalized k-space.

For a more detailed analysis of the behavior of the designed metalenses, we focused on the broadband and single-wavelength designs with NA = 0.8 and *f* = 90 µm at $\lambda$ = 1.55 µm, whose phase profiles are shown in Fig. 3(e,b) (blue dashed line). We also considered an ideal metalens having exactly the target phase profile shown in Fig. 3(e,b) with solid orange lines. We assumed a plane wave illuminating the metalenses with different angles and we computed the distribution of the electric field right after the three metalenses described above. To this purpose, we considered both the phase delay and the transmission efficiency of each meta-atom as included in the prepared library and shown in Fig. 2(b,e) at $\lambda$ = 1.55 µm. For the ideal metalens, we assumed that each meta-atom had unitary transmission efficiency. We then exploited Fourier analysis to study the outgoing fields. The resulting spectra in the spatial frequency domain for the three metalenses along the normalized $k_x/k_0$ axis (with $k_0$ the wave vector in vacuum) are reported in Fig. 9. Complete 2D spectra are shown in Fig. S6 of the supplementary information document. Figure 9(a-c) show with blue lines the normalized spatial spectrum for the broadband metalens for three different incident angles of $0^{\circ }$, $30^{\circ }$, and $43.8^{\circ }$. Figure 9(d-f) show with blue lines the same series of results but for the single-wavelength metalens for illuminations at $0^{\circ }$, $30^{\circ }$, and $89^{\circ }$. Normalized spatial frequencies for both broadband and single-wavelength metalenses are limited to $|k_x/k_0| < \pi /p/k_0 = 1.2$, determined by the metasurface period *p* = 650 nm. Red lines in each panel report the results for the ideal metalens for the corresponding illumination angle. Black vertical dashed lines mark the propagation region in k-space, defined as the range of spatial frequencies that can propagate in free space. Beyond this area, waves become evanescent with a purely imaginary z-axis wave vector $k_z$. The propagation condition can be written as:

4 $$\begin{aligned} \sqrt{k_x^2 + k_y^2} < k,\ \text {with}\ k = n_f k_0 \end{aligned}$$

where $n_f$ is the refractive index in the focusing region (with $n_f = 1$ in our case). It should be noticed that the spatial spectrum of a metalens depends on its numerical aperture and in our case a value of NA = 0.8 or larger ensures non-zero components (at least in the ideal case) across the entire $k_x/k_0$ propagation range [−1,1] (for normal incidence), as it is shown also in Fig. S7 of the supplementary information document.

For the ideal metalens with an incident plane wave normal to the metasurface (Fig. 9(a,d), red lines) the normalized spatial spectrum has its maximum at $k_x=0$ and its Fourier components maintain as expected non-zero values across the entire [−1,1] range. This spatial spectrum closely resemble that of the single-wavelength metalens, Fig. 9(d), blue line. On the contrary, the spectrum of the broadband metalens, Fig. 9(a), blue line, is essentially concentered around $k_x=0$, with nearly zero amplitude for different $k_x$ values, despite the design numerical aperture was maintained at NA = 0.8. This difference can be attributed to the noisier phase profile that resulted from the design procedure described in Sec. Design and that can be seen in Fig. 3(e).

Tilting the angle of the incident plane wave causes the spectra to shift in the k-space as shown in Fig. 9(b,c,e,f), indicating a change of propagation direction that ultimately causes the transversal shift of the focal spot. When the angle of the incident wave is tilted by $30^{\circ }$, Fig. 9(b,e), spectra shift toward positive $k_x/k_0$ values of about $\Delta k_x(\theta ) = n_i/n_f\ sin(\theta )$ = 0.72. Despite the shift, the single-wavelength metalens (as well as the ideal one) still maintain a largely non-zero spatial spectrum across the propagation region, ensuring the quality of the focal spot is maintained also with tilted illumination, see for example Fig. 5(f). The broadband metalens show instead a more asymmetrical spectrum, causing the emergence of stronger off-axis aberrations^27^ which tends to degrade the quality of the focal spot. This can be observed in Fig. 5(c) for an illumination tilting of $21^{\circ }$.

Finally, when the incident angle is larger than $43.8^{\circ }$, Fig. 9(c), the amplitude of the spatial spectrum for the broadband metalens within the propagation region becomes negligible. As a result, there is no more light propagating in the free space and the metalens reaches the limit of the field of view. This is what can be observed in the experimental results shown in Fig. 5(c) for an illumination tilting of $43^{\circ }$. The focal spot is deformed and could not be observed for larger illumination angles. As before, the broader spatial spectrum of the single-wavelength design ensures the focal spot is formed across the entire field of view up to a $90^{\circ }$ tilting of the illuminating plane wave, Figs. 9(d) and 5(f). The difference between the spectrum of the ideal metalens and the single-wavelength one comes from the different sampling steps (650 nm for the single-wavelength metalens and 220 nm for the ideal phase profile), which cause different periodicity and aliasing in the Fourier domain. The impact on the Fourier spectrum of the sampling steps is further discussed in Fig. S8 of the supplementary information document.

## Discussions and conclusion

In summary, we have demonstrated a single-layer metalens with a broadband achromatic behavior and a wide field of view. By leveraging a library of waveguide-like silicon meta-atoms with diverse wavelength-dependent phase delays, we successfully engineered the dispersion of a quadratic phase profile. This achievement represents a significant advancement over prior approaches, which often rely on doublet metalens designs, multi-layer meta-atoms, or hybrid geometric–propagation phase methods. The experimental characterization across the 1.5 µm – 1.6 µm bandwidth (limited by our setup) showed focusing up to a field of view of $\pm 43^{\circ }$ and a relative focal length shift as low as 1.3%, an order-of-magnitude reduction compared to a conventional quadratic metalens used as a reference. As a consequence of the reduced chromatic aberration, focusing efficiency also remained constant in the considered wavelength range.

Furthermore, we exploited Fourier analysis to understand the impact of dispersion engineering on the metalens field of view. Our investigation suggests that the need to simultaneously match a target phase profile and its dispersion using the available library of meta-atoms introduced additional phase noise that shrunk the spatial spectra of the broadband metalens, causing a reduction of the field of view from the theoretical $180^{\circ }$ limit. The limit predicted by the analysis ($\pm 43.8^{\circ }$) was indeed in a very good agreement with the value measured experimentally. A possible way to address this limitation could be expanding the meta-atom design space using more diverse geometries, potentially guided by machine learning or optimization techniques. Such an approach could reduce phase noise, better match the target phase profile, and further improve both achromatic and field-of-view performance.

The combination of broadband achromaticity and wide angular performance that we have demonstrated, achieved through a single-layer design fully compatible with standard nanofabrication processes, offers a practical and scalable alternative to complex multi-layer architectures for integrating metalenses into photonic systems, particularly for beam steering applications in the near-infrared, offering also a promising pathway toward broadband and wide field of view metalenses for imaging systems operating in the visible range.

## Method

### Metalenses fabrication

Metalenses have been realized in the cleanroom of the Centre de Nanosciences et de Nanotechnologies using e-beam lithography with positive resist (ZEP) and proximity effect correction. After development (AR-600-546), structures have been etched with inductively coupled plasma reactive ion etching (ICP RIE). The sample has been finally cleaned using butanone, piranha solution, and oxygen plasma.

### Determination of the focal distance

To determine the relative focal length of the metalenses, we first determined the focus x and y coordinates by searching for the maximum intensity. We then extracted the (normalized) intensity pattern along the z-axis at the focal spot position and we fitted it with a Gaussian function whose center we took as the z-coordinate of the focus. The relative focal length was then determined as the difference between the focal length at $\lambda$ = 1.55 µm and the focal length at the considered wavelength. It should be noticed that, for a given metalens, all the characterizations for different wavelengths and tilting angles have been performed without changing the position of the sample, hence ensuring the same origin for the axes.

## Supplementary Information

Supplementary Information.

## Data Availability

The datasets generated and analyzed during the current study are available in the Zenodo repository: https://doi.org/10.5281/zenodo.16919981.
