# Supplementary material for: Single-layer silicon metalens for broadband achromatic focusing and wide field of view
Source: Sci Rep. 2025 Dec 8;15:43343. doi: 10.1038/s41598-025-27208-1 (PMC12686017; doi:10.1038/s41598-025-27208-1)
Supplement: Supplementary file 1 — Supplementary Information. [file 41598_2025_27208_MOESM1_ESM.pdf]

SINGLE-LAYER SILICON METALENS FOR BROADBAND ACHROMATIC FOCUSING AND WIDE FIELD OF VIEW: SUPPLEMENTARY INFORMATION DOCUMENT

Jian Cao, Sarra Salhi, Jonathan Peltier, Jean-René Coudevylle, Samson Edmond, Cédric Villebasse, Etienne Herth, Laurent Vivien, Carlos Alonso-Ramos, Daniele Melati

Centre de Nanosciences et de Nanotechnologies, Université Paris-Saclay, CNRS, 91120 Palaiseau, France

Table S1. Experimental results reported in the literature for achromatic and/or wide field-of-view metalenses. FOV: Field of view. NA: Numerical aperture. Norm. relative focal shift: ratio between the change of the focal distance in the considered wavelength range and the designed focal distance. Focusing efficiency: (*) the ratio between the power in the focal spot region and the incident power; (+) the ratio between the power in the focal spot region and the transmitted power through metalens area. Reference numbers are at the end of this document. References are reported at the end of this document.

FOV(°)	Norm. relative focal shift	Wavelength	NA	Focusing efficiency	Type	Ref.
\	~0.15%	488 - 658 nm	0.70	~15%*	Singlet	[1]
\	~12.9%	400 - 660 nm	0.11	30% - 67%*	Singlet	[2]
\	7.2%	1200 – 1600 nm	0.12	21.2%-51.4%*	Singlet	[3]
\	5.2%	1300 – 1600 nm	0.85	45.7%-55.7%*	Singlet	[3]
>170°	\	5.2 μm	0.24	32% - 45%*	Singlet (Pinhole)	[4]
≈180°	\	940 nm	0.2	41% - 88%*	Singlet (Pinhole)	[4]
>170°	\	532 nm	0.80	25%+	Singlet	[5]
86°	\	1500 - 1640 nm	0.83	~ 7.9%+	Singlet	[6]
<10°	~0.1%	488 - 658 nm	0.70	~12%	Singlet	[7]
\	9.3%	470 – 670 nm	0.20	~20%*	Singlet	[8]
\	5.4%	1000 – 1800 nm	0.06	~65.6%(average)*	Hybrid	[9]
50°	\	632 nm	0.71	~9%*	Doublet	[10]
86°	1.3%	1500 - 1600 nm	0.80	~ 20%+	Singlet	This work

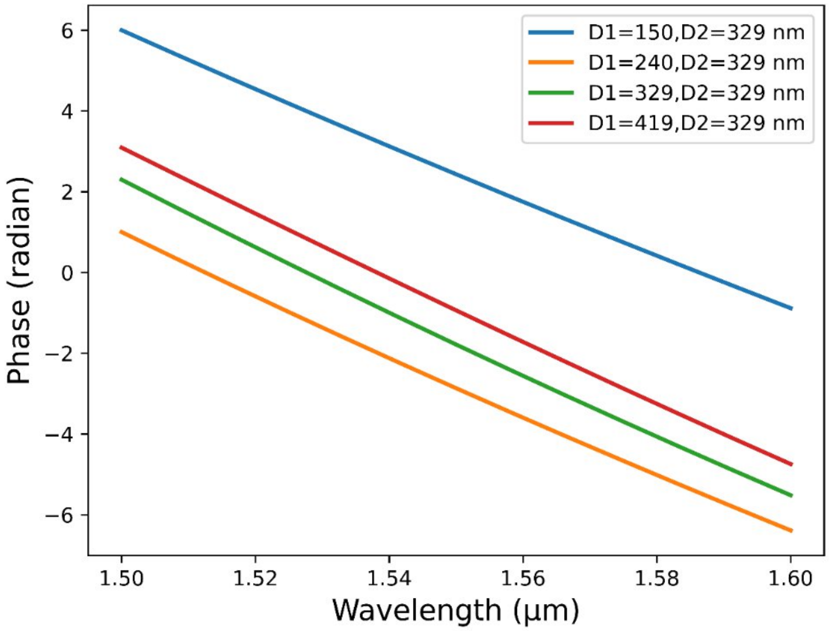

Fig. S1. Phase delay imparted by four meta-atoms with different shapes (chosen as examples) as a function of wavelength in the studied wavelength range from 1.5 µm to 1.6 µm. using five sampling points allows matching in a robust way the dependence of the imparted phase delay on wavelength.

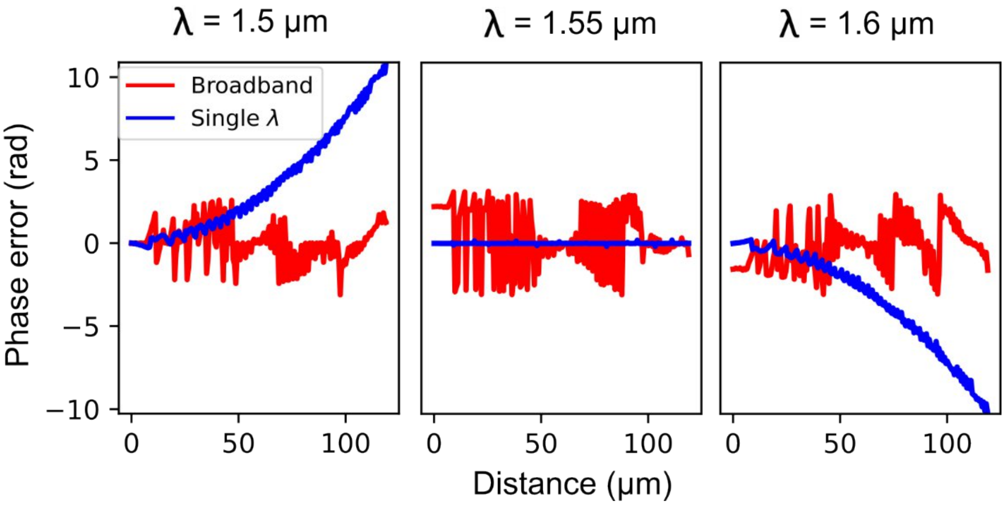

Fig. S2. Phase error for a metalens with a numerical aperture of 0.8, focal distance of 90 µm and radius of 120 µm at three different wavelengths in the 1.5 µm – 1.6 µm range. The phase error is calculated as the difference between the target phase delay and the meta-atom phase delay for a given wavelength and for each position along a radius of the metalens. Red solid lines refer to the broadband metalens while blue solid line to the single wavelength one.

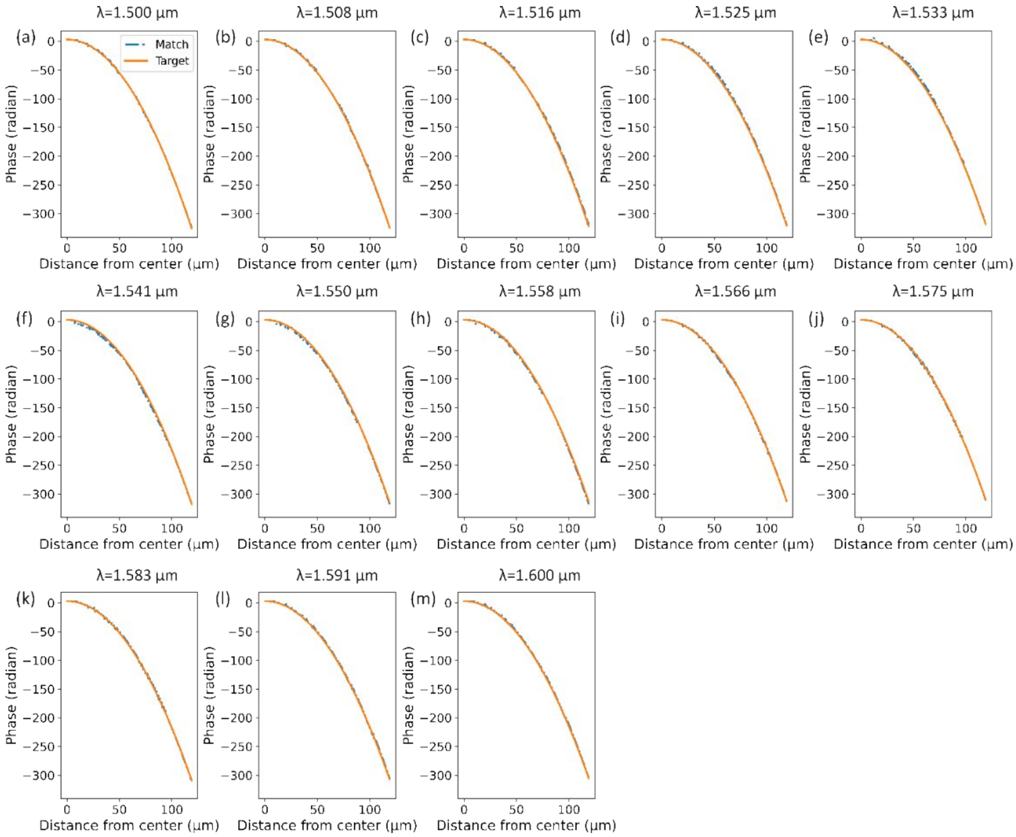

Fig. S3. Phase profile matching results for the broadband design metalens for several wavelength points in the 1.5 μm to 1.6 μm range. The blue dotted lines indicate the matched phase profile along the diameter of the metalens. The orange solid lines indicate the target phase profile.

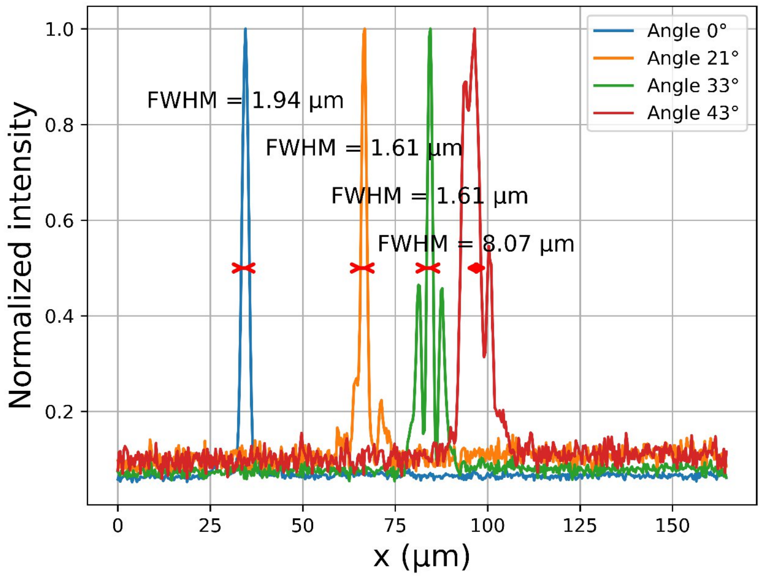

Fig. S4. Full width at half maximum (FWHM) of the focal spot for the broadband design metalens studied in this work at different incident angles from 0° (normal incidence) to 43° and at a wavelength of 1.55 µm. The FWHM is measured along the x-axis (see Figure 5(c) in the manuscript). The FWHM remains almost constant (1.61 µm - 1.94 µm) until the illumination angle approaches 43°, which is the theoretical limit of the filed of view for the broadband design described in this work. Focusing performance degenerates sharply for $\theta_i = 43°$ (FWHM = 8.07 µm) and beyond this angle a focal spot could not be observed.

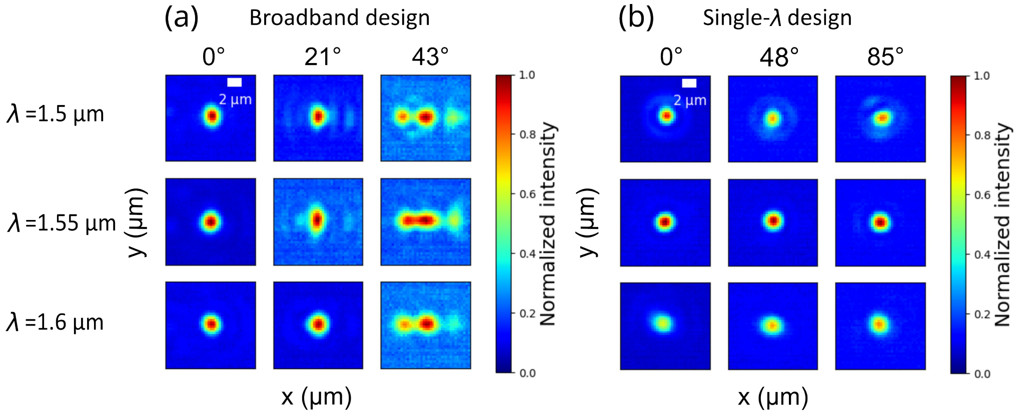

Figure S5. Experimental characterization of the metalens focusing for (a) the broadband and (b) single wavelength metalenses. Compared to Figs. 5(c) and (f) in the manuscript, images here are taken at a fixed distance from the metalens instead of at the center of the focal spot. In particular, images are taken at a distance equal to the focal distance at λ = 1550 nm. Because of chromatic aberration, the spot goes out of focus for the single wavelength metalens upon wavelength shift.

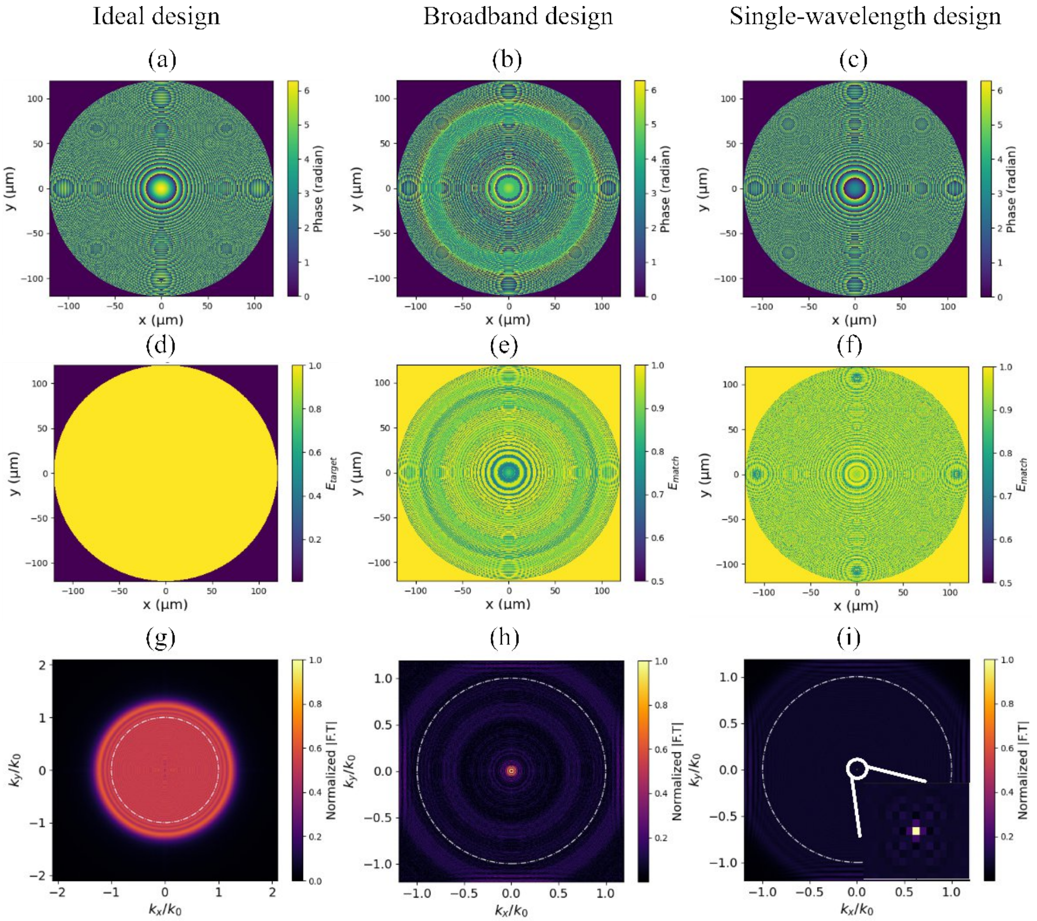

Fig. S6. Fourier transform results for (a,d,g) ideal, (b,e,h) broadband and (c,f,i) single-wavelength metalenses, with numerical aperture of 0.8, radius of 120 μm at λ = 1.55 μm. (a) – (c) Phase profile of metalenses. (d) – (f) Normalized amplitude of the electric field after the metalenses. (g) – (i) Normalized 2D Fourier transform results in k-space. The white circle denotes the propagation region.

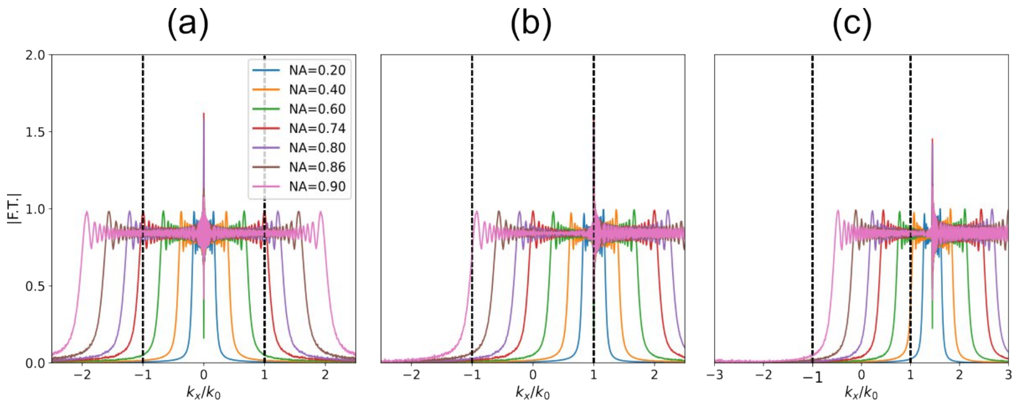

Fig. S7. 1D normalized spatial spectra along the k_x/k_0 axis for an ideal metalens implementing exactly the target phase profile shown in Fig. 9 of the manuscript for $\lambda = 1.55$ µm, a radius of 120 µm, and numerical aperture varying from 0.2 to 0.9. (a) Spatial spectrum for normal illumination $\theta = 0°$, (b) illumination tilted at $\theta = 43.8°$, and (c) at $\theta = 89°$

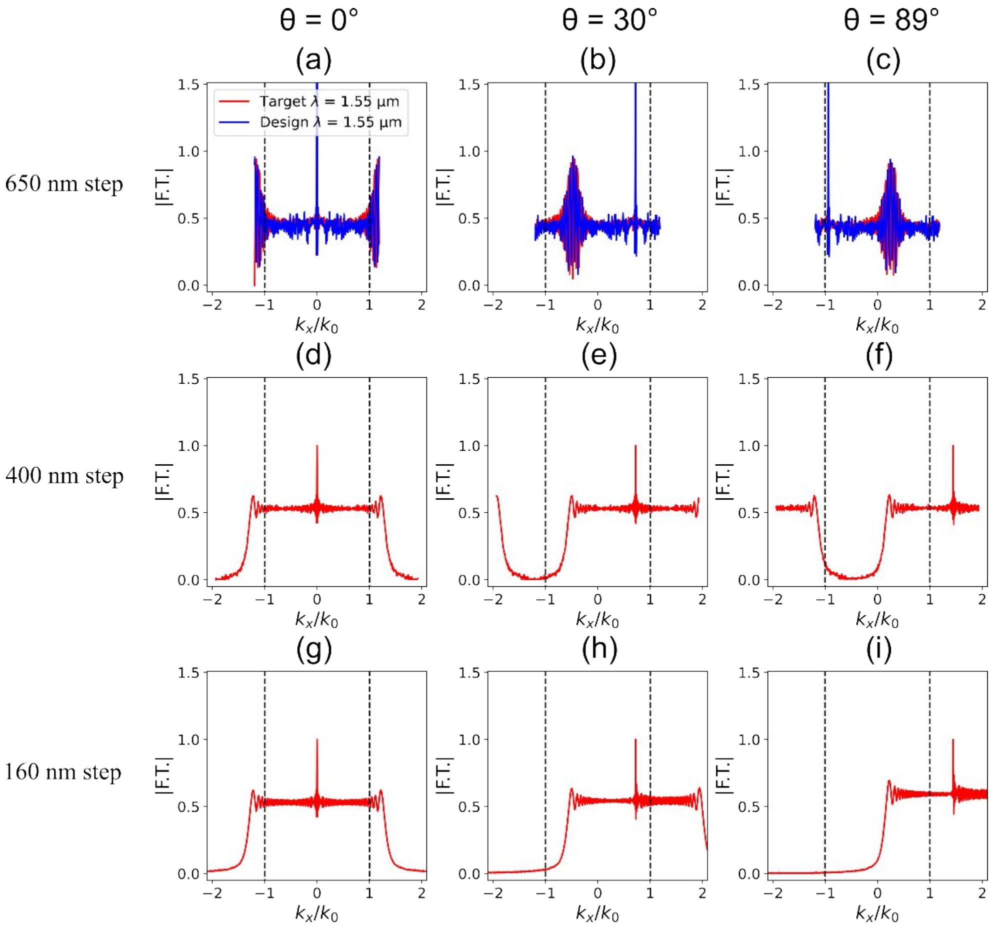

Fig. S8. 1D normalized spatial spectra of the transmitted electric field for metalenses with a numerical aperture of 0.8, a radius of 120 μm, and with different sampling steps of the electric field. (a) - (c) Results for a sampling step of 650 nm, as determined by the period of the fabricated metalenses, at three different incident angle in the range 0° - 89°. The red solid lines refer to the spectra of the ideal metalens while the blue solid lines to that of the single-wavelength metalens. (d) – (f) The same series of results with a sampling step of 400 nm. and (g) – (i) for a sampling step of 160 nm. The latter are the ones reported in the manuscript.

References

[1] Li Z, Pestourie R, Park J S, et al. Inverse design enables large-scale high-performance meta-optics reshaping virtual reality[J]. Nature communications, 2022, 13(1): 2409.

[2] Wang S, Wu P C, Su V C, et al. A broadband achromatic metalens in the visible[J]. Nature nanotechnology, 2018, 13(3): 227-232.

[3] Shrestha S, Overvig A C, Lu M, et al. Broadband achromatic dielectric metalenses[J]. Light: Science & Applications, 2018, 7(1): 85.

[4] Shalaginov M Y, An S, Yang F, et al. Single-element diffraction-limited fisheye metalens[J]. Nano Letters, 2020, 20(10): 7429-7437.

[5] Martins A, Li K, Li J, et al. On metalenses with arbitrarily wide field of view[J]. Acs Photonics, 2020, 7(8): 2073-2079.

[6] Liu Y, Zhang J, Le Roux X, et al. Broadband behavior of quadratic metalenses with a wide field of view[J]. Optics Express, 2022, 30(22): 39860-39867.

[7] Li Z, Lin P, Huang Y W, et al. Meta-optics achieves RGB-achromatic focusing for virtual reality[J]. Science Advances, 2021, 7(5): eabe4458.

[8] Chen W T, Zhu A Y, Sanjeev V, et al. A broadband achromatic metalens for focusing and imaging in the visible[J]. Nature nanotechnology, 2018, 13(3): 220-226.

[9] Balli F, Sultan M, Lami S K, et al. A hybrid achromatic metalens[J]. Nature communications, 2020, 11(1): 3892.

[10] Li Z, Wang C, Wang Y, et al. Super-oscillatory metasurface doublet for sub-diffraction focusing with a large incident angle[J]. Optics Express, 2021, 29(7): 9991-9999.
